# The Role of Mitochondrial Protein UPS1 in Regulating Pathogenicity of Candida albicans

**DOI:** 10.3390/jof12060411

**Published:** 2026-06-04

**Authors:** Qianwen Xu, Changlong Xie, Dinghui Wang, Xiaoxiao Zhu, Wenfan Wei, Xiaojia Niu, Tianming Wang, Hongchen Wang, Daqiang Wu

**Affiliations:** 1 Department of Pathogenic Biology and Immunology, College of Integrated Chinese and Western Medicine, Anhui University of Chinese Medicine, 350 Longzihu Road, Hefei 230012, China; 3440711135@stu.ahtcm.edu.cn (Q.X.); wenfanwei2022@163.com (W.W.); wtm1818@163.com (T.W.); 2 Hefei Institute of Pharmaceutical Industry Co., Ltd., 10 Wenshan Road, Hefei 230037, China

**Keywords:** *Candida albicans*, UPS1, mitochondria, hyphal formation, biofilm, pathogenicity

## Abstract

The mitochondrial membrane protein UPS1, a conserved intermembrane space protein in *Saccharomyces cerevisiae*, possesses phosphatidic acid transfer activity and plays a positive regulatory role in processes such as cardiolipin metabolism and transport. The role of UPS1 protein in pathogenic fungi such as *Candida albicans* has not been explored, especially in relation to its influence on virulence factors like hyphal growth and biofilm formation, which are crucial for the pathogenicity of *C. albicans*. The research investigated the function of the UPS1 protein in *C. albicans* by using gene knockout techniques, analyzing mitochondrial function, and conducting tests for hyphal and biofilm development. The results revealed that deletion of the *UPS1* gene leads to altered mitochondrial morphology, increased reactive oxygen species levels, and reduced intracellular ATP content, thereby causing severe growth defects in *C. albicans*. In addition, transcriptomic analysis indicated that loss of *UPS1* significantly represses the expression of genes associated with hyphal growth and biofilm formation. Functional assays further confirmed that *UPS1* deficiency markedly impairs cell adhesion capability, hyphal development, and biofilm formation of *C. albicans*. Notably, deletion of the UPS1 protein markedly reduces the susceptibility of *C. albicans* to membrane-targeted antifungal drugs. Finally, infection models using *Galleria mellonella* larvae and a murine vulvovaginal candidiasis model verified that *UPS1* gene knockout attenuates the pathogenicity of *C. albicans*. In summary, our findings demonstrate that UPS1 protein modulates the pathogenicity of *C. albicans* by regulating mitochondrial function, hyphal growth, and biofilm formation.

## 1. Introduction

*Candida albicans*, a prevalent conditional pathogenic fungus, is known to cause mucocutaneous and systemic candidiasis, posing a great global health threat [1,2]. Under physiological conditions, *C. albicans* exist as a commensal organism, coexisting harmoniously with the host’s microbial flora without inducing disease. Nevertheless, upon host immunosuppression or dysregulation of the intestinal microbiota, *C. albicans* shifts to a pathogenic phenotype and elicits invasive infectious processes [3]. Endowed with diverse virulence factors and strong environmental adaptability, *C. albicans* can colonize multiple ecological niches within the host. Well-characterized virulence-associated traits include the ability to undergo yeast-to-hyphal morphological transition, expression of cell surface adhesins, and assembly of biofilms [4]. Given that the pathogenicity of *C. albicans* is closely linked to its ability to adapt to host microenvironments and regulate virulence factor expression, deciphering the molecular mechanisms underlying these processes is critical for combating *C. albicans* infections.

As core organelles in eukaryotes, mitochondria are indispensable for energy production via cellular respiration and oxidative phosphorylation [5], and they also serve as key regulatory hubs in fungal cells, orchestrating biological processes closely associated with *C. albicans* pathogenicity—including nutrient metabolism, biofilm development, drug resistance, and virulence [6]. Accumulating evidence indicates that mitochondria play a pivotal role in the pathogenesis of fungal pathogens, including *C. albicans*, *Aspergillus fumigatus*, and *Cryptococcus neoformans*, particularly during adaptation to host-derived stresses such as nutrient limitation, oxidative stress, and immune surveillance [7,8]. Our research team previously reported that *OCT1* (a mitochondrial protein quality control protease) and Num11 (a mitochondrial anchor protein) modulate mitochondrial function in *C. albicans*, thereby regulating the formation of virulence factors like hyphae and biofilms and ultimately influencing in vivo pathogenicity [9,10]. Notably, following synthesis in the endoplasmic reticulum, phosphatidic acid (PA) must be transported across the outer mitochondrial membrane to the inner mitochondrial membrane (IMM), a process mediated by the mitochondrial Ups1/Mdm35 protein family [11]. Thus, investigating the biological functions of mitochondria-associated lipid transport proteins in pathogenic fungi like *C. albicans* is crucial for understanding fungal pathogenesis and developing novel antifungal strategies.

UPS1 is a member of the evolutionarily conserved UPS1/PRELI protein family in Saccharomyces cerevisiae and localizes to the mitochondrial intermembrane space [12]. Specifically, the heterodimeric complex formed by Ups1/Mdm35 in yeast facilitates PA transport from the endoplasmic reticulum to the IMM, where PA is further converted into cardiolipin (CL) a signature phospholipid of mitochondria. Loss of Ups1/PRELID1 proteins disrupt CL biosynthesis, thereby impairing mitochondrial structure and function [13]. Initially identified as a regulator of Mgm1 (a yeast homolog of human OPA1 essential for mitochondrial fusion), Ups1 deficiency in yeast reduces the short isoform of Mgm1 (s-MEFgm1) and disrupts tubular mitochondrial cristae [14]. Additionally, *UPS1* deletion decreases CL levels, while simultaneous knockout of Ups1 and its homolog Ups2 restores CL content. Subsequent studies confirmed Ups1’s role as a lipid transfer protein mediating PA transport between mitochondrial membranes [3]. However, despite its well-characterized function in S. cerevisiae, UPS1’s role in pathogenic fungi like *C. albicans* and its potential regulatory effects on *C. albicans* pathogenicity-related traits remain largely unexplored.

Our team constructed the *UPS1* deletion strain (*ups1*∆/∆) and the rescue strain. (*ups1*∆/∆: *UPS1*) to systematically analyze *UPS1*’s impact on *C. albicans*’ mitochondrial function, carbon source utilization, and temperature adaptability. Transcriptome sequencing combined with qRT-PCR validation was used to explore its molecular regulatory mechanisms, while in vitro virulence factor assays and in vivo infection models verified its role in *C. albicans* pathogenicity. This research aims to clarify *UPS1*’s biological function and regulatory mechanisms in *C. albicans*, providing a theoretical basis for anti-Candida infection studies.

## 2. Materials and Methods

### 2.1. C. albicans Strains and Growth Conditions

The strain information in this study is detailed in Table S1. The *C. albicans* strains were routinely cultured at 30 °C using liquid medium and solid medium (YPD medium). After revival, the strains were stored in 50% glycerol solution at −80 °C and subsequently revived by solid plate culture on YPD medium at 30 °C.

### 2.2. Strains’ Construction

Leucine (LEU), histidine (HIS), and arginine (ARG) auxotroph are characteristics of strain SN152, with the strain deficient in the biosynthesis of these three amino acids. In the present study, the *ups1*Δ/Δ deletion mutant and the complementary strain *ups1*Δ/Δ*: UPS1* were successfully constructed using the HIS1-LEU2-ARG4 auxotrophic system combined with CRISPR-Cas9 gene knock-in technology. All primers used for strain construction are listed in Table S2, and the corresponding plasmid information is summarized in Table S3.

The *HIS1-LEU2-ARG4* auxotrophic strategy is widely used for sequential *C. albicans* gene knockout due to its dual marker flexibility [15,16]. The HIS1 cassette (from plasmid pSN52) and *UPS1* homologous arms were fused by PCR and transformed into SN152; the LEU2 cassette (from plasmid pSN40) was used similarly to delete the second *UPS1* allele. For CRISPR-Cas9 editing, a split-marker strategy with NAT resistance selection was adopted. For *UPS1* complementation, the LEUpOUT CRISPR system was used: MssI-linearized gRNA plasmid, CAS9 plasmid (PADH139), and *UPS1* fragment were co-transformed, selected on YPD + NAT plates, and CRISPR elements/NAT marker were removed via -LEU counter-selection. LEU^+^/NAT^−^ phenotype verification confirmed successful *UPS1* complementation [17].

### 2.3. Subcellular Co-Localization Analysis of UPS1 Protein with Mitochondria

To verify the subcellular localization characteristics of UPS1 protein, *C. albicans* wild-type strains carrying the UPS1-mNeonGreen fusion tag were inoculated into YPD liquid medium and cultured overnight. The fungal suspension was diluted to a concentration of 1 × 10^6^ cells/mL using sterile PBS. The diluted fungal suspension was mixed with a 200 μM Mito Tracker Red working solution at a ratio of 1:1000 under light protection and incubated for 30 min in a 30 °C incubator under light protection. After two PBS washes, mitochondrial structures were observed using the Leica laser scanning confocal microscope (Leica, Wetzlar, Germany) at an excitation wavelength of 579 nm.

### 2.4. Mitochondrial Morphology

Assessment of mitochondrial morphology involved Mito-Tracker Red staining followed by microscopic imaging [18]. The specific operation was as follows: the overnight-cultured *C. albicans* suspension was adjusted to a concentration of 1.0 × 10^6^ cells/mL with phosphate-buffered saline (PBS), mixed with 200 μM Mito Tracker Red working solution at a ratio of 1:1000, and incubated in a 30 °C constant temperature incubator in the dark for 30 min. After two washes with PBS, the mitochondrial structure was observed at an excitation wavelength of 579 nm using a Stellaris 5 Cryo (Leica, Wetzlar, Germany).

### 2.5. Measurement of Intracellular ATP Contents

This research used the BacTiter-Glo™ Microbial Cell Viability Assay Kit (Promega Corporation, Madison, WI, USA) to quantitatively measure intracellular ATP levels and assess the impact of *UPS1* deletion [19]. Overnight incubation of strains was done in YPD medium, and the Fungal broth was standardized to 1.0 × 10^7^ cells/mL with PBS. Cell suspension and BacTiter-Glo reagent were combined in equal amounts and incubated for 5 min. The relative light unit (RLU) signal was measured using a multifunctional microplate reader with full wavelength capability.

### 2.6. Measurement of Mitochondrial Membrane Potential (MMP)

MMP level was determined using the JC-1 MMP detection kit (Beyotime Biotechnology, Shanghai, China) [20]. The pre-cultured fungal suspension was adjusted to 1.0 × 10^7^ cells/mL, with an equal volume of JC-1 stain added thereafter [21]. After gentle and thorough mixing, the mixture was incubated at 37 °C in the dark for 20 min. Following two rounds of washing with PBS, the fluorescence intensities at 590 nm and 530 nm were measured simultaneously by a flow cytometer(Becton Dickinson, Franklin Lakes, NJ, USA), and the red-to-green fluorescence ratio was subsequently calculated. Meanwhile, image acquisition was performed using a Leica laser scanning confocal microscope (Leica, Germany) laser scanning confocal microscope with consistent wavelength settings.

### 2.7. Measurement of Intracellular Reactive Oxygen Species Levels

Intracellular reactive oxygen species (ROS) levels were determined using a ROS detection kit (Beyotime, Shanghai, China). Briefly, the fungal suspension was adjusted to 1.0 × 10^6^ cells/mL, and DCFH-DA was added to a final concentration of 10 μM. The mixture was incubated at 30 °C for 1 h in the dark. After washing twice with sterile PBS, fluorescence signals were visualized under a fluorescence microscope (Olympus Corporation, Tokyo, Japan) with excitation at 488 nm and emission at 525 nm [21]. Subsequently, fluorescence intensity was quantitatively analyzed using ImageJ 1.8.0 software (version 1.8.0; National Institutes of Health, Bethesda, MD, USA) [22].

### 2.8. Susceptibility Assays

First, the cultured strains were adjusted to a concentration of 1.0 × 10^6^ cells/mL. After which, 10-fold serial dilutions were performed to reach a final concentration of 1.0 × 10^1^ cells/mL. 5 μL of serially diluted suspensions were spotted onto Solid media supplemented with various types of carbon sources or stress-inducing agents, followed by incubation at 30 °C, 35 °C, and 37 °C for 48 h, respectively. For the carbon source utilization experiments, cells were diluted to 300 cells/mL and inoculated onto both fermentable (YPD) and non-fermentable (YPEG:3% Ethanol, 3% Glycerol) culture media. After incubation at 30, 35, and 37 °C for 48 h, the diameters of colonies were measured using a vernier caliper. Three non-fermentable carbon sources (YPE: Ethanol, YPC: Citrate, YPG: Glycerol) and two fermentable carbon sources (YPD, YPM: Maltose) were used. Membrane stress was induced by Synthetic Complete medium (SC), 0.05 μg/mL amphotericin B (AmB), 2 μg/mL fluconazole (FLU), and 0.04% Synthetic Dextrose Medium (SDS) [23]. 50 μg/mL Congo red (CR) and 200 μg/mL calcofluor white (CFW) were used to induce cell wall stress in fungal cells [24].

### 2.9. Growth Curve Measurement

The strains were cultured in YPD medium until the stationary phase. The cells were washed with PBS and adjusted to an initial OD_600_ value of 0.04. The resulting cell suspension was incubated at 30 °C, 200 rpm for 24 h, and the OD_600_ values were recorded at 2 h intervals throughout the incubation period. The doubling time (DT) during the logarithmic growth phase was calculated by employing the following formula: DT = t × ln2/ln (Nt/N_0_), where Nt denotes the cell density at time, and N_0_ stands for the initial cell density [25].

### 2.10. RNA-Seq Analysis

Samples were prepared through the overnight cultivation of all strains in YPD liquid medium at a temperature of 30 °C. The overnight cultures were subsequently transferred to fresh YPD medium to continue their growth, and cells were collected during the logarithmic growth phase (with an OD_600_ value of 0.8). The pretreated *C. albicans* sample was taken and added with 1 mL Trizol solution on ice. After fully vortexed and mixed, it was allowed to stand on ice for 5 min. Then 1 mL chloroform was added. After vortexed and mixed on ice for 5 min, it was centrifuged at 12,000 rpm for 15 min in a 4 °C centrifuge. The product after centrifugation was divided into three layers. After carefully collecting the uppermost water layer into a new microcentrifuge tube, an equivalent volume of isopropanol was added, followed by vigorous vortex homogenization. The mixture was kept on ice for 10 min and then centrifuged at 12,000 rpm and 4 °C for 10 min. Abandon the supernatant; after pre-cooling on ice with 75% ethanol prepared with DECP water, 1 mL of washing precipitate was added to each precipitate group under centrifugation. The precipitate was centrifuged at 12,000 rpm at 4 °C for 10 min to discard the supernatant. After standing at room temperature and volatilizing ethanol, 100 μL of DEPC water was added to re-suspend RNA, which was sub-packed and stored at – 80 °C. Three samples were repeated in each group. A Nanodrop2000 instrument (Thermo Fisher Scientific, Waltham, MA, USA) was employed to assess the RNA concentration and purity of each sample. The concentration, purity and integrity of RNA in each group were detected to ensure that the concentration of RNA samples was up to standard, the ratio of OD_260_/OD_280_ was between 1.8 and 2.0, and there was no protein or genomic DNA pollution, which met the requirements of high-throughput sequencing experiments. Subsequently, cDNA library construction and transcriptome sequencing were performed on the Illumina NovaSeq 6000 platform (Illumina, San Diego, CA, USA). The cDNA library serves as the essential sequencing template, and all raw transcriptomic data are directly acquired by sequencing this library, which facilitates the screening of differentially expressed genes and the analysis of overall transcriptional characteristics of *C. albicans* [26].

### 2.11. Quantitative Real-Time PCR

The strains were incubated to the logarithmic growth phase (OD_600_ = 0.8). RNA was isolated using the SPARKeasy Yeast RNA Extraction Kit (Sparkjade, Jinan, China) (3 samples/group). For vaginal inflammatory factor transcription, mouse vaginal tissue RNA was extracted via the Trizol method (3 samples/group) [27]. RNA was reverse transcribed into cDNA. qRT-PCR was performed with cDNA templates, β-actin as internal reference, results analyzed by the 2^−ΔΔCT^ method; primers are in Supplementary Table S4.

### 2.12. Filamentation Assay

First, the activated strains were adjusted to a cell concentration of 1.0 × 10^5^ cells/mL. The adjusted *C. albicans* suspension was then plated onto various culture media plates, including YPD, YPD supplemented with 10% FBS, Lee’s medium, Spider medium, and SLAD medium. 5 µL aliquot of each suspension was dripped onto the corresponding plates, which were then incubated at 37 °C for 7 days. Subsequently, hyphal formation was observed using six liquid media that are known to induce hyphal growth, namely RPMI-1640, YPD, YPD with 10% FBS, Lee’s, Spider, and SLAD media [28]. For this assay, we normalized *C. albicans* suspension was incubated at 37 °C for 4 h, followed by microscopic observation of hyphal formation.

### 2.13. Biofilm Production Assay

To evaluate biofilm synthesis, cells were incubated with shaking for 12–14 h, pelleted, and resuspended in RPMI 1640 to achieve 1.0 × 10^6^ cells/mL. For adhesion assays, 100 µL of culture medium with an appropriate strains concentration was added to the 96-well plate, followed by incubation at 37 °C for 90 min. After incubation, wells were rinsed with PBS to remove non-adherent cells. The supernatant was aspirated, followed by three gentle washes with PBS, and then 150 µL of XTT solution was added under light protection. Following a 3 h incubation at 37 °C, the absorbance of each well was recorded at 490 nm using a microplate reader (Thermo Fisher Scientific, Waltham, MA, USA) [29]. The calcofluor white (CFW) staining method was also used to observe the process of biofilm formation, and the specific experimental procedures were as follows: First, the strains were cultured overnight in YPD liquid medium. Next, adjust the concentration of *C. albicans* to 1.0 × 10^6^ cells/mL and add it to RPMI-1640 medium for cultivation. The adjusted cell suspension was incubated at 37 °C for 4 h, followed by gentle washing with PBS 3 times. After washing, 1 mL of RPMI-1640 medium was added, and the samples were statically placed in a 37 °C incubator for 24 h. The cells were then washed with PBS. Subsequently, 50 µg/mL CFW was added to the samples for staining. Finally, biofilm formation was visualized and observed under a Leica laser scanning confocal microscope (Leica Microsystems, Wetzlar, Germany) with appropriate magnification [30].

### 2.14. In Vitro Adhesion Assay

To investigate the impact of *UPS1* gene deletion on vitro adhesion in *C. albicans*, fetal bovine serum (FBS) was added to 12-well plates, followed by overnight incubation at 37 °C with shaking at 75 rpm. Spider liquid medium was employed to standardize the fungal suspension of each group to an OD_600_ of 0.5. The FBS was then removed from the 12-well plates, which were washed with PBS before inoculation with the fungal suspension. The plates were further incubated at 37 °C with shaking at 150 rpm for 48 h. Finally, non-adherent cells were removed by washing with PBS before observation.

### 2.15. Galleria Mellonella Infection Assay

For assessing how *UPS1* gene deletion affects the in vivo infection of *C. albicans*, 64 susceptible larvae with a body length of 2.0–2.5 cm were chosen to conduct the virulence evaluation [31]. PBS was used to adjust the strains to a standardized concentration of 8.0 × 10^6^ cells/mL, followed by the division of *G. mellonella* larvae into four separate groups: the blank control group (only inject PBS as the negative control group), the wild type, the *ups1*∆/, and the *ups1*∆/∆: *UPS1*. Each larva in the four groups received an injection of 10 µL fungal inoculum into the left or right suboesophageal ganglion utilizing a fine-tipped microinjector. After 48 h of incubation, three larvae were randomly selected from each group, ground, and inoculated onto solid YPD plates. The plates were inverted and incubated at 30 °C for 48 h to analyze fungal load of each group. Additionally, the mortality of *G. mellonella* larvae was monitored every day, and continuous observation was performed for 14 days to generate the survival curve.

### 2.16. Study on Experimental Vulvovaginal Candidiasis (VVC) in Mice

A mouse model of vulvovaginal candidiasis (VVC) was established following the protocol reported previously [32]. To investigate the impact of *UPS1* gene deletion on local in vivo infection, a total of 48 C57BL/6 mice, each weighing 20 ± 2 g, were used in this study. All mice were subjected to a 7-day acclimatization period in a controlled environment, where the temperature was maintained at 23 ± 2 °C and the relative humidity was kept between 40% and 70%. During the adaptation and experimental periods, mice were freely provided with standard pellet feed and drinking water. All animal care and management practices were performed in strict compliance with the ethical guidelines approved by the Animal Ethics Committee of the Chinese Center for Disease Control and Prevention, as well as the standards specified in the Guide for the Care and Use of Laboratory Animals. After a week of adaptive culture, the mice were randomly divided into four groups: control group, wild-type group, knockout group, and reconstitution group. On days 1, 3, and 5 of the experiment, each mouse was subcutaneously injected with 0.1 mL of 0.1 mg/mL estradiol benzoate, which was dissolved in edible oil and sterilized before use. The day after the final injection, obvious vaginal epithelial shedding was observed, indicating that the mice were in a pseudo-pregnant state. Subsequently, for 7 consecutive days, mice in the control group received intravaginal administration of PBS, whereas those in the other groups were inoculated intravaginally with a *C. albicans* suspension at a concentration of 2 × 10^7^ cells/mL to establish vaginal infection.

At Day 7 post-infection, mouse vaginas were rinsed with PBS. A 20 μL aliquot of lavage fluid was spread onto a glass slide, dried, and stained with crystal violet for 1 min, iodine for 1 min, decolorized with 95% ethanol for 30 s, and counterstained with safranin for 30 s. The stained slides were observed under a light microscope (Olympus, Tokyo, Japan). A 50 µL aliquot of the rinse was plated on solid YPD medium and incubated at 30 °C for 48 h. Subsequently, visual observation and quantitative analysis of the fungal load in each experimental group were performed. On the last day of infection, the vaginas of the mice were gently rinsed with PBS to collect the lavage fluid. A 20 μL aliquot of the collected lavage fluid was evenly spread onto a glass slide, followed by air-drying at room temperature. The slide was then subjected to Gram staining sequentially: stained with crystal violet for 1 min, treated with iodine for 1 min, decolorized with 95% ethanol for 30 s, and counterstained with safranin for 30 s. After the staining process was completed, the stained slides were observed under a light microscope to examine the infection-related morphological features.

### 2.17. Enzyme-Linked Immunosorbent Assay

For the quantitative assessment of the fungal pathogen’s capacity to trigger vaginal inflammation in mice, the researchers employed three enzyme-linked immunosorbent assay (ELISA) kits, namely Mouse Tumor Necrosis Factor-α (TNF-α), Mouse Interleukin-6 (IL-6), and Mouse Interleukin-10 (IL-10) ELISA kits (Beyotime, Shanghai, China). Mouse blood was centrifuged at 4 °C and 3000 rpm for 20 min. After collecting the serum, the detection was carried out in strict accordance with the instructions provided by the ELISA kits. The experiment included adding standards and samples to a 96-well plate, followed by incubation with the enzyme solution for 60 min. Subsequently, the plate was washed thoroughly, chromogen solution and stop solution were added sequentially, and the absorbance was measured at 450 nm using a microplate reader.

### 2.18. Statistical Analysis

All data in this experiment were derived from three independent biological replicates and presented as mean ± SD. Comparative analysis of data between the two groups was carried out via Student's *t*-test, while GraphPad Prism 9.0 software (GraphPad, San Diego, CA, USA) was used for all statistical analyses, with a significance threshold of *p* < 0.05.

## 3. Results

### 3.1. C. albicans UPS1 Exerts a Critical Role in Mitochondrial Homeostasis

To characterize the function of the *UPS1* protein in mitochondrial homeostasis, a *ups1*∆/∆deletion mutant was constructed via homologous recombination using the HIS1-LEU2-ARG4 auxotrophic system, and a corresponding revertant strain *ups1*∆/∆*: UPS1* was generated via CRISPR-Cas9-mediated complementation. Successful construction of all strains was verified by auxotrophic screening and polymerase chain reaction (PCR) identification (Figure S1).

**Figure 1:**
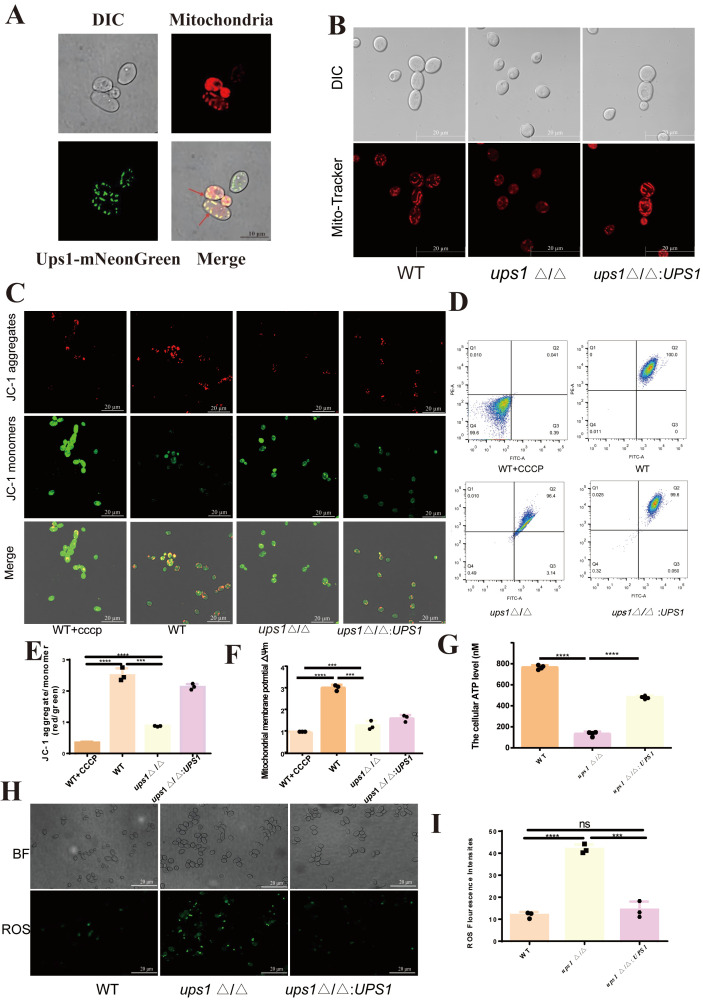
*UPS1* gene deletion impairs mitochondrial function in *C. albicans*. (**A**) Subcellular localization of Ups1. The strain expressing Ups1-mNeonGreen was stained with MitoTracker Red to label mitochondria. The yellow signal in the merged panel indicates co-localization of Ups1-mNeonGreen (green) with mitochondria (red), confirming that Ups1 localizes to mitochondria in *C. albicans*. Scale bar: 10 μm. (**B**) Compared with the wild-type group, the knockout strain showed a mitochondrial structure fragmentation phenotype. Scale: 20 μm. (**C**) Representative images of JC-1 staining in WT, *ups1*Δ/Δ, and complemented strains. JC-1 aggregates (red) indicate high mitochondrial membrane potential, while JC-1 monomers (green) indicate low potential. Scale bar: 20 μm. (**D**) MMP membrane potential fluorescence imaging detected by Confocal microscopy. (**E**) Analysis of JC-1 membrane potential fluorescence intensity,JC-1 aggregate/monomer (red/green). (**F**) The average red and green fluorescence intensity ratio of each group was measured. (**G**) The ATP content of the test strains. (**H**) The level of reactive oxygen species. Scale: 20 μm. (**I**) The quantification comparison of fluorescence distribution of each strain. Statistically significant markers in this figure and following figures are as follows: ns, no significant difference; ***, *p* < 0.001; ****, *p* < 0.0001.

*UPS1* is a member of the evolutionarily conserved UPS1/PRELI protein family in Saccharomyces cerevisiae and is predominantly localized to the mitochondrial intermembrane space. In this study, the mNeonGreen fluorescent tag was fused to the UPS1 protein to characterize its subcellular localization via fluorescence localization assay. Figure 1A fluorescence observation revealed that the fluorescent signal of the UPS1-mNeonGreen fusion protein exhibited obvious colocalization with mitochondrial fluorescence signals. In the figure, the arrow points to the area distinctly yellowed, which corresponds to the co-localization site of the UPS1 protein with mitochondria. Collectively, these findings demonstrate that UPS1 protein is closely associated with mitochondria in *C. albicans*.

Mitochondrial phenotypes were subsequently compared among the wild-type (WT), *ups1*∆/∆mutant, and complemented strains. Mitochondrial morphology was visualized using Mito-Tracker Red fluorescence staining. As shown in Figure 1B, differential interference contrast (DIC) imaging revealed no overt alterations in general cellular morphology among the three strains. In fluorescence microscopy, WT cells displayed predominantly punctate and rod-shaped mitochondria with intact structure, whereas the *ups1*∆/∆mutant exhibited severe mitochondrial fragmentation. Mitochondrial morphology in the *ups1*∆/∆*: UPS1* revertant was restored to a pattern comparable to that of the WT strain.

Under normal conditions, the inner mitochondrial membrane maintains a highly negative potential (mitochondrial transmembrane potential, ΔΨm). JC-1 accumulates in mitochondria in a concentration-dependent manner driven by the potential difference, thereby altering its fluorescence color. When mitochondria are intact with a high membrane potential, substantial amounts of JC-1 enter the mitochondria and form J-aggregates, emitting red fluorescence. In contrast, when the mitochondrial membrane potential declines or undergoes depolarization, JC-1 is released from mitochondria into the cytoplasm and exists in monomeric form, producing green fluorescence. The level of mitochondrial membrane potential is evaluated by detecting the fluorescence intensity ratio of red to green. A higher ratio indicates elevated membrane potential and normal mitochondrial function, while a lower ratio represents reduced membrane potential and mitochondrial damage [33,34]. The *ups1*Δ/Δ strain displayed attenuated red fluorescence and enhanced green fluorescence, and this phenotypic defect was rescued in the complementary strain. These results demonstrate that the *ups1*Δ/Δ strain presents a marked reduction in mitochondrial membrane potential accompanied by moderate mitochondrial damage. In addition, mitochondrial membrane potential (ΔΨm) was assessed via JC-1 staining (Figure 1C), flow cytometry (Figure 1D) and fluorescence detection. Under the condition that the cell number remained relatively consistent in each field, ImageJ software was applied to calculate the ratio of red to green fluorescence intensity based on three independent biological replicates. The average value was calculated to obtain quantitative results (Figure 1E). Quantitative analysis clearly demonstrates that in the positive control group WT-CCCP, under conditions of complete mitochondrial damage, the red fluorescence is markedly weak while the membrane potential level shows a significant decrease. In the WT group, the red fluorescence is strong, the green fluorescence is weak, resulting in a higher fluorescence ratio and elevated membrane potential levels, indicating normal mitochondrial function. In contrast, the *ups1*∆/∆ group exhibits reduced red fluorescence intensity, decreased red-to-green fluorescence ratio, lowered membrane potential, and mitochondrial damage; whereas the *ups1*∆/∆: *UPS1* group shows a notable recovery in membrane potential levels. With CCCP-treated WT cells as a positive control for complete depolarization. Flow cytometry was performed using identical cell numbers for each group. Fluorescence intensity measurement was applied to distinguish cellular states of different fungal strains. Figure 1D revealed that in the CCCP treatment group, cells distributed in the Q2 region accounted for merely 0.04%, whereas Q4 cells reached 99.6%. This distribution pattern reflected decreased mitochondrial membrane potential. Nearly all wild-type cells were in the Q2 region (high ΔΨm), suggesting elevated membrane potential levels and normal mitochondrial function. The ups1Δ/Δ strain exhibited similar membrane potential reduction to the CCCP group (96% Q2 compared to WT), confirming severe depolarization and mitochondrial damage. The complemented strain restored Q2 cells to 99.6%, consistent with WT. As presented in Figure 1G, WT cells maintained an ATP level of approximately 820 nM, while the *ups1*∆/∆mutant displayed drastically reduced ATP concentration of around 135 nM. The ATP content in the complemented strain recovered to approximately 477 nM, which was significantly higher than that of the deletion mutant. These results indicate that *UPS1* exerts a pronounced regulatory effect on mitochondrial ATP synthesis. In addition, as depicted in Figure 1H,I, the fluorescence intensity representing ROS accumulation was approximately 14 in WT cells but rose significantly to around 38 in the *ups1*∆/∆ strain. ROS levels in the complemented strain returned to approximately 16, like the WT. These observations demonstrate that deletion of *UPS1* leads to excessive intracellular ROS accumulation. Collectively, these findings from multiple perspectives establish that *UPS1* plays an indispensable role in preserving mitochondrial functional homeostasis in *C. albicans*.

### 3.2. UPS1 Deficiency Impacted C. albicans Growth

Given the essential contribution of *UPS1* to mitochondrial function, we further explored its role in supporting cellular growth and metabolic fitness. Energy and nutrient metabolism are fundamental to all biological processes, in which mitochondria serve as the central regulatory hub [35]. The carbon metabolism pathways of *C. albicans* mainly include glycolysis and oxidative phosphorylation, and mitochondrial dysfunction is typically accompanied by impaired respiratory metabolism [36].

**Figure 2:**
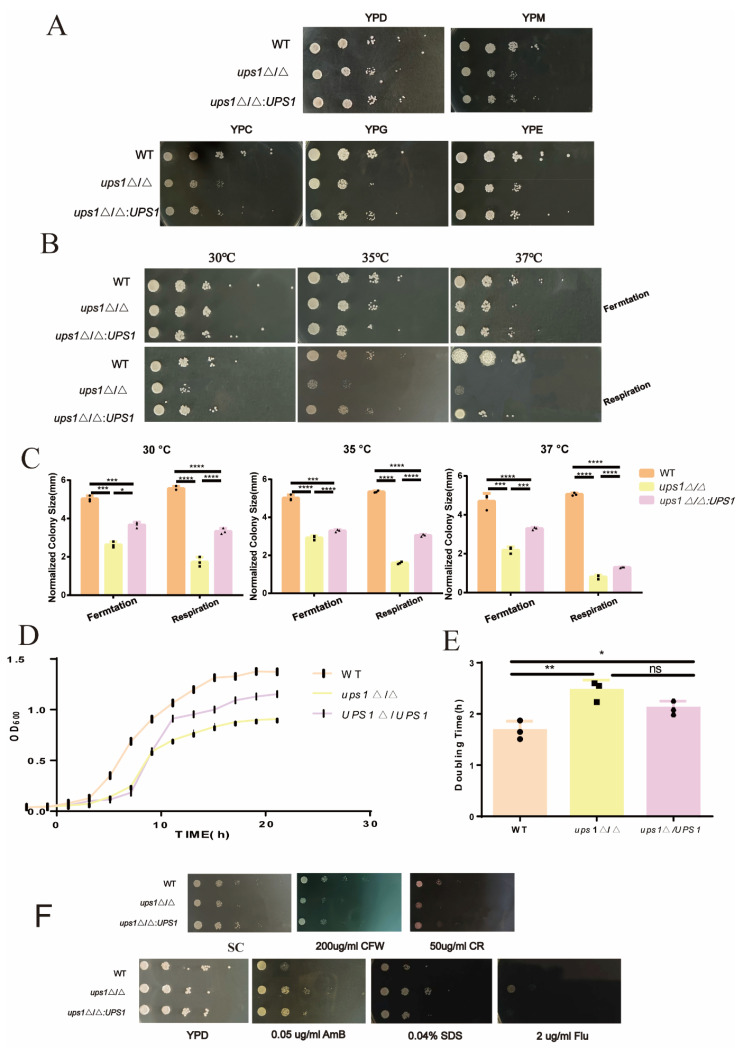
*UPS1* affects the growth ability of *C. albicans*. (**A**) Growth phenotypes of the indicated strains in media containing fermentable versus nonfermentable carbon sources. (**B**,**C**) The growth patterns of each strain under aerobic and anaerobic conditions at different temperatures. (**D**,**E**) The 24 h growth curve and logarithmic growth phase doubling time of each strain were analyzed. (**F**) Drop plate experiments of different cell wall pressure reagent medium and different cell membrane pressure reagent medium of each strain. Statistically significant markers in this figure and following figures are as follows: ns, no significant difference; *, *p* < 0.05; **, *p* < 0.01; ***, *p* < 0.001; ****, *p* < 0.0001.

To define the role of *UPS1* in carbon source utilization, growth phenotypes were assessed on media containing fermentable (YPD, YPM) and non-fermentable carbon sources (YPC, YPG, YPE). As shown in Figure 2A, the *ups1*∆/∆ mutant exhibited markedly restricted colony expansion relative to the WT strain under all tested conditions, whereas the complemented strain showed obvious growth recovery. To investigate how temperature influences strain growth, three strains were cultivated at 30 °C, 35 °C, and 37 °C in media containing either fermentable or non-fermentable carbon sources. Growth phenotypes are presented in Figure 2B,C. In glucose rich fermentable conditions, the colony morphology of the three strains showed no obvious temperature-dependent alterations. In contrast, when grown on respiration medium with glycerol and ethanol as non-fermentable carbon sources, the *ups1*∆/∆ mutant exhibited increasingly severe growth defects relative to the WT strain as the temperature rose. At 37 °C, the *ups1*∆/∆ strain was almost unable to grow. These results demonstrate that deletion of *UPS1* in *C. albicans* impairs the ability to utilize glycerol and ethanol, suggesting that mitochondrial respiratory function is significantly compromised in the knockout strain, particularly at elevated temperatures. The colony size was quantified by measuring the diameter of the first colony in each dilution series from the spot dilution assays shown in Figure 2B. To ensure consistency, the diameter of a well-isolated, representative colony from the highest-density spot (first dilution) was measured using ImageJ software, with three independent biological replicates analyzed for each condition. The normalized colony size was calculated and presented in Figure 2C. The results are shown in Figure 2C: the *ups1*∆/∆ strain exhibited significantly smaller colony diameters compared to the WT group, and under anaerobic conditions, the colonies of the knockout strain were markedly smaller than those of the aerobic group. Additionally, growth capacity declined markedly with increasing temperature.

In addition to energy production, intracellular carbon sources also serve as important components for constructing structures such as the cytoskeleton and cell wall. Furthermore, studies have shown that different carbon sources can affect the stress tolerance of fungal cells. We initially graphed the growth curves of various strains to examine the impact of *UPS1* on cell proliferation. Figure 2D illustrates. The strains were cultured in YPD liquid medium at 30 °C with 300 rpm shaking for 24 h. The biomass concentration in the medium was measured every 2 h using the optical density (OD) method. The *ups1*∆/∆ strain exhibited significantly reduced proliferation compared to both the WT strain and the *ups1*∆/∆*: UPS1* complemented strain. In addition, we compared the time required for the biomass of different strains to double (Figure 2E). The logarithmic growth phase of the growth curve (8–12 h) was selected to calculate the doubling times of each strain using the specific growth rate formula μ = (lnOD_2_ − lnOD_1_)/(t_2_ − t_1_) and the doubling time formula Td = 0.693/μ. Growth kinetics of the wild-type (WT), *ups1*Δ/Δ, and complemented strains were assessed by monitoring OD_600_ over 24 h (Figure 2D). Consistent with spot dilution assays, the *ups1*Δ/Δ mutant showed significantly impaired growth, with a slower OD_600_ increase during the exponential phase; the complemented strain partially restored this defect. To quantify these differences, doubling defined as the time for the population to double in number—was calculated (Figure 2E). The WT doubling time was 1.8 h, while the *ups1*Δ/Δ mutant exhibited a significantly prolonged doubling time (2.5 h), indicating a severe growth rate reduction. The complemented strain showed partial recovery (2.2 h), the growth rate has recovered.

We assessed the sensitivity of the three strains to cell wall stress reagents and antimicrobial agents. We add CR and CFW to the medium to induce cell wall stress in the strains. Fluconazole (FLU) and amphotericin B (AmB), both antifungal agents targeting the cell membrane, were used alongside sodium dodecyl sulfate (SDS) to induce cell membrane stress. The results (Figure 2F) showed that the *ups1*∆/∆mutant strain did not exhibit an abnormally sensitive phenotype under the action of the cell wall stress agents CFW and CR, but showed a certain degree of tolerance to FLU, AmB, and SDS. The *UPS1* gene appears to play a role in the regulation of the cell membrane and drug resistance in *C. albicans*.

### 3.3. Transcriptomic Analysis of UPS1 Function

**Figure 3:**
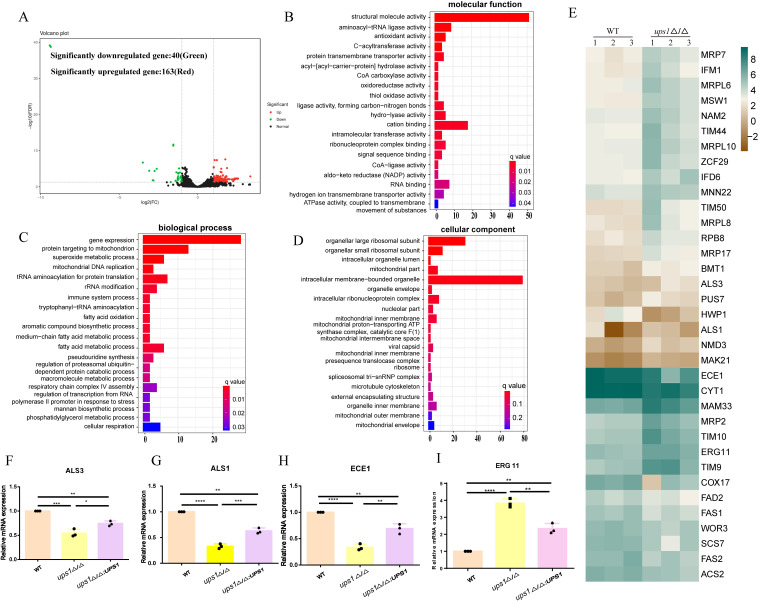
RNA sequencing analysis uncovers the potential functional implications of *UPS1*. (**A**) A volcano plot was generated to present the significantly differentially expressed genes (DEGs) in the *ups1*∆/∆ strain. (**B**–**D**) Gene Ontology functional enrichment analysis was performed to exhibit the main distribution of upregulated and downregulated DEGs in the *ups1*∆/∆ strain, which were categorized into molecular function, biological process, and cellular component, respectively. (**E**) Hierarchical clustering was conducted on the screened DEGs to cluster genes that share identical or similar expression profiles. (**F**–**I**) Expression of virulence-related genes in different strains. Statistically significant markers in this figure and following figures are as follows: *, *p* < 0.05; **, *p* < 0.01; ***, *p* < 0.001; ****, *p* < 0.0001.

To explore the regulatory role of *UPS1* at the transcriptional level, RNA-seq was performed in the *ups1*∆/∆mutant and the parental WT strain, with reads mapped to the *C. albicans* reference genome. As summarized in Figure 3A and Table S5, a total of 163 genes were significantly upregulated, and 40 genes were significantly downregulated in the mutant relative to the WT, using thresholds of |log_2_(fold change)| > 1 and FDR < 0.05. Gene Ontology (GO) enrichment analysis was conducted to classify these differentially expressed genes (DEGs) according to molecular function (MF), cellular component (CC), and biological process (BP) (Figure 3B–D). The q-value, calculated via the Benjamini–Hochberg procedure, is the false discovery rate (FDR)-adjusted *p*-value used to control for multiple testing bias in gene ontology (GO) enrichment analysis. It quantifies the probability that an observed enrichment event is a false positive. In the enrichment plots (Figure 3B–D), the color gradient encodes q-values, where red indicates smaller q-values (higher enrichment significance), and purple indicates larger q-values (lower significance). Terms with smaller q-values (structural molecule activity, intracellular membrane-bounded organelle, and protein targeting mitochondrion) represent the most statistically reliable and biologically relevant functional categories affected by *UPS1* deletion [37]. Functional annotation revealed that *UPS1* is predominantly involved in enzymatic activities and multiple core cellular processes, including mitochondrial protein targeting, mitochondrial DNA replication, and respiratory metabolism. It also contributes to gene expression, tRNA modification, stress responses, and metabolic homeostasis.

In addition, *UPS1* also exerts an important influence on the synthesis of several key cellular components, including the small and large subunits of ribosomes, ribonucleoprotein complexes, the lumens of various organelles, certain membrane structures, and some mitochondrial structures. The results suggest that *UPS1* is essential for mitochondrial function maintenance. We also organized the identified significantly differentially expressed genes, excluding those that had not been named. As shown in Figure 3E. All gene expression levels in the heatmap were normalized relative to the wild type (WT). The color bars indicate the expression changes in the *ups1*Δ/Δ mutant compared to WT: green represents genes with upregulated expression in the mutant, while brown represents genes with downregulated expression. The comparison between the two datasets in the figure is based on the WT. This heatmap includes not only numerous genes related to mitochondrial function (such as *MRP2*, *MRPL6*, *MRP7*, *MRPL37*, *TIM23*, and *TOM6*) but also genes associated with hyphae and cell wall synthesis (*ECE1*, *MNN22*, *ZCF29*, *HWP1*, *BMT1*), genes involved in drug resistance and biofilm synthesis *(ALS1*, *ALS3*, *HWP1*, *ACO1*, *ALK8*, *WOR3*), and additionally, genes related to copper ion metabolism (COX17, COD6). These results suggest that *UPS1* may play an important role in the aforementioned cellular processes. Furthermore, we utilized real-time quantitative PCR (qPCR) to measure the transcriptional levels of selected genes associated with the defective phenotype of the *ups1*∆/∆ strain. The *ECE1*, *ALS1*, and *ALS3* genes are essential for the virulence of *C. albicans*. Specifically, *ECE1* participates in the processes of biofilm formation, adhesion, and filamentation. In contrast, *ALS1* and *ALS3*—members of the agglutinin-like sequence (ALS) gene family—facilitate the pathogenicity of *C. albicans* and its adhesion to vaginal mucosal and epithelial cells. Figure 3F–I show that the *UPS1∆/∆*strain exhibits downregulated expression of three mycelium-associated genes compared to the wild-type strain. Specifically, *ALS1* was downregulated by approximately 2.658-fold, *ALS3* by approximately 1.735-fold, and *ECE1* by approximately 2.773-fold. In contrast, the gene *ERG11*, which serves as a primary target for azole antifungal agents, showed a significant upregulation, increasing by approximately 3.67-fold. Since *ALS3* and *ECE1* are typically not transcribed independently in YPD medium, their expression is primarily induced under mycelial growth conditions. We cultured the strains in RPMI1640 medium until the logarithmic growth phase, collected the strains to extract RNA, and subsequently performed reverse transcription quantitative polymerase chain reaction (RT-qPCR) analysis. The results are shown in Figure S3, demonstrating that the knockout strain *ups1*∆/∆ exhibited downregulated expression of both *ALS3* and *ECE1* genes, with more pronounced effects. The findings confirm that *UPS1* plays a role in modulating *C. albicans*’ adhesion, aggregation, and hyphal growth, alongside a decrease in drug sensitivity. qPCR experiments (Figure 3F–I): All qPCR data are expressed as the mean ± standard deviation (SD) of at least three biological replicates. Differences among wild-type (WT), ups1Δ/Δ mutant strains, and complementary strains were analyzed using one-way ANOVA and Tukey’s multiple comparison test.

### 3.4. The Absence of UPS1 Hinders Hyphal Development, Adhesion, and Biofilm Formation in C. albicans

**Figure 4:**
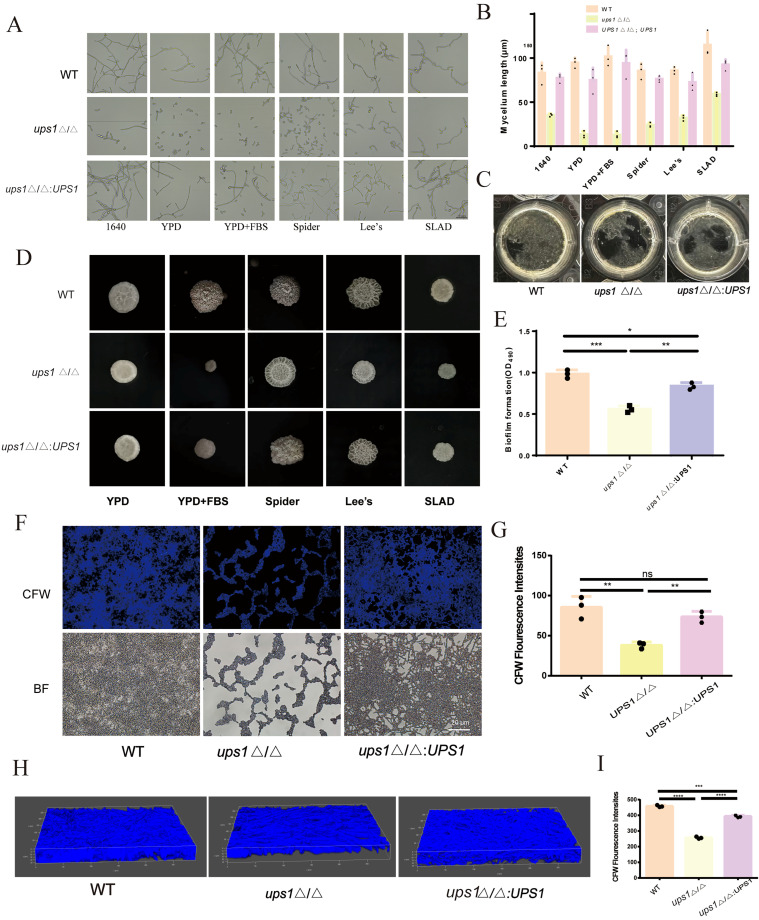
Deletion of *UPS1* gene in *C. albicans* affects its ability to form hyphae, develop biofilms and surface adhesion. (**A**) The hyphal formation ability of *C. albicans* in liquid medium. (**B**) Quantitative Analysis of Liquid Mycelial Length. (**C**) In vitro adhesion test results of wild type, *ups1*∆/∆ and *ups1*∆/∆: *UPS1* strains. (**D**) The hyphal formation ability of *C. albicans* on solid medium. Scale: 4 mm. (**E**) XTT reduction assay for detecting biofilm formation capability. (**F**) The biofilm formation ability of *C. albicans* was observed by fluorescence microscope. Scale: 20 μm. (**G**) Quantitative analysis of fluorescence images of each strain was performed using ImageJ software. (**H**) Leica confocal microscope was used for three-dimensional imaging observation. Scale: 100 μm. (**I**) Quantitative analysis of the fluorescence images of each strain was performed using ImageJ software. Statistically significant markers in this figure and the following figures are as follows: ns, no significant difference; *, *p* < 0.05; **, *p* < 0.01; ***, *p* < 0.001; ****, *p* < 0.0001.

Based on the previous transcriptomic analysis results, we gained insights into the potential functions that *UPS1* may be involved in. Consequently, we analyzed the *ups1*∆/∆strain’s abilities in hyphal formation, biofilm formation, and adhesion. We examined the hyphal formation of the WT strain, *ups1*∆/∆mutant strain, *ups1*∆/∆*: UPS1* complemented strain in six liquid media: RPMI-1640, YPD, YPD + 10% FBS, Lee’s, Spider, and SLAD (Figure 4A). To quantify hyphal morphogenesis, three independent images were acquired for each strain, and hyphal length was measured using ImageJ software with scale-bar calibration (Figure 4A). Mean hyphal length was calculated per image, and all experiments were performed in biological triplicate (Figure 4B). Across all inducing conditions, the wild-type strain exhibited vigorous budding and polarized hyphal extension. Conversely, the *ups1Δ/Δ* mutant was profoundly impaired in filamentation, producing only truncated pseudo hyphae and failing to elaborate mature hyphae. Reintroduction of *UPS1* largely restored the wild-type pattern of filamentous growth in the complemented strain. To further clarify the growth defects of the *UPS1* knockout mutant, another group was observed for hyphal growth in liquid medium after 2 h incubation. As shown in Figure S2, the knockout strain group exhibited only yeast-like morphology and minimal pseudo hyphal growth even in hyphal induction medium, which contrasted sharply with the wild-type strain that formed well-developed, elongated hyphae. Analysis of adhesion ability (Figure 4C) revealed that the *ups1*∆/∆mutant exhibited significantly reduced adhesion compared to the wild-type strain.

We also evaluated the colony morphology of the strains across five solid media (Figure 4D). Compared with the WT strain, the colony diameter of the *ups1∆/∆*strain was significantly reduced in all media, and the colony edges were unable to form normal hyphae. The *ups1*∆/∆ *UPS1* complemented strain exhibited colony morphology akin to the wild-type strain. We examined *UPS1*’s impact on *C. albicans* biofilm formation utilizing the XTT assay and Calcofluor White staining (Figure 4E–I). Figure 4G,I present quantitative analysis of the biofilm fluorescence images using ImageJ software. The results indicated that the *ups1*∆/∆strain exhibited markedly decreased biofilm formation compared to the WT, with notable deficiencies in both density and thickness. The findings suggest that *UPS1* is crucial for adhesion, hyphal and biofilm formation.

### 3.5. Abrogation of the UPS1 Gene Causes a Marked Decrease in the Pathogenicity of C. albicans

**Figure 5:**
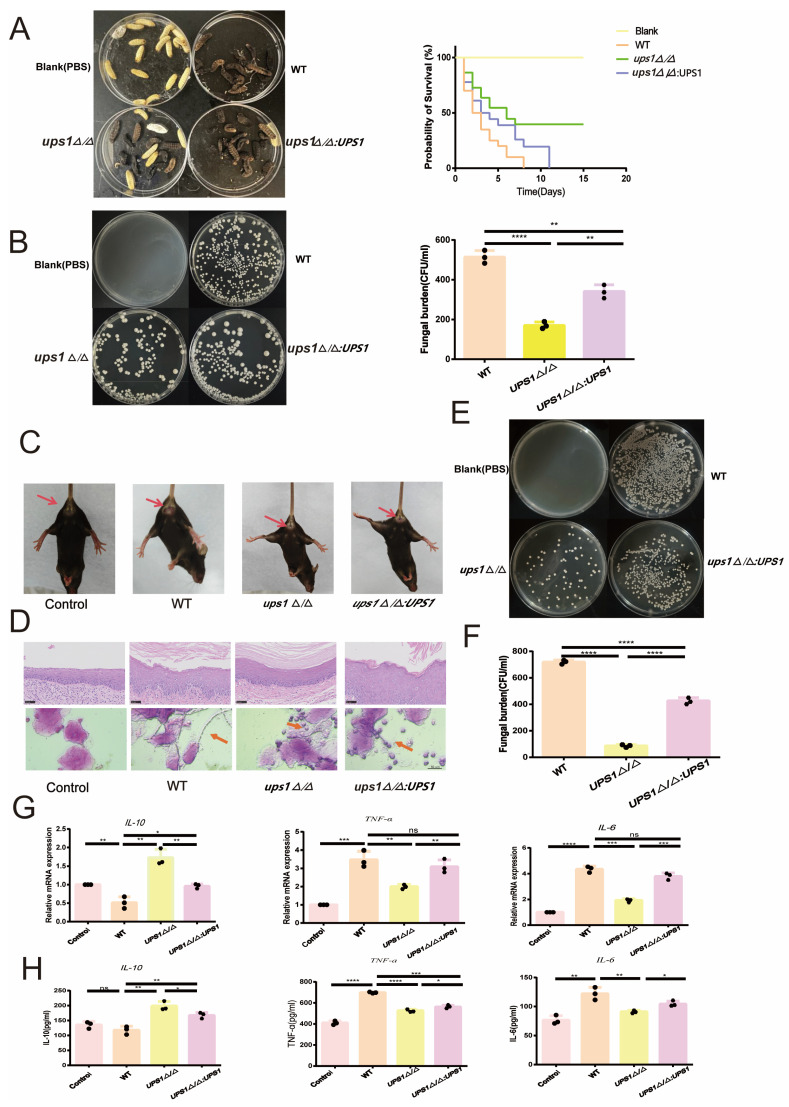
*UPS1* deficiency diminishes the virulence and infectivity of *C. albicans* in both *G. mellonella* larvae and mice. (**A**) The survival rate of *C. albicans* infected wax moth. (**B**) Fungal load of *C. albicans* infected wax moth. (**C**) Vaginal local map of mice infected with *C. albicans*, the red arrow points to the vaginal region of the mouse. (**D**) Vaginal H & E staining (scale: 100 μm), vaginal lavage fluid Gram staining (scale: 50 μm), the red arrow indicates the mycelial portion in the irrigation solution. (**E**,**F**) Fungal load in vaginal lavage fluid of mice infected with *C. albicans*. (**G**) Expression level of vaginal inflammatory factors in mice infected with *C. albicans*. (**H**) The content of vaginal inflammatory factors in mice infected with *C. albicans*. Statistically significant markers in this figure and the following figures are as follows: ns, no significant difference; *, *p* < 0.05; **, *p* < 0.01; ***, *p* < 0.001; ****, *p* < 0.0001.

Given the regulatory roles of *UPS1* in filamentation, adhesion, and biofilm formation, we evaluated its contribution to *C. albicans* virulence. Therefore, we investigated the effect of the *UPS1* gene on the virulence of *C. albicans.* First, we used an invertebrate model, *G. mellonella* larvae, to analyze the virulence of the *ups1*∆/∆strain. seventy-six *G. mellonella* larvae with similar growth status were randomly divided into groups, then infected with cells of WT, *ups1*∆/∆ and *ups1*∆/∆ *UPS1*, and their survival status was recorded. The findings (Figure 5A) indicate that by the 7th day, all larvae in the wild-type infection group had perished. In contrast, the *ups1*∆/∆infection group maintained a 40% survival rate from the 6th day onward, It remained unchanged even 14 days after the final day of the experiment. while all larvae in the *ups1*∆/∆*: UPS1* group succumbed to the 11th day. In addition, 48 h after infection, *G. mellonella* larvae were randomly selected and there in vivo fungal load was detected (Figure 5B). The *UPS1∆/∆*group larvae exhibited a significantly lower fungal load compared to the wild-type group, whereas the *ups1*∆/∆*: UPS1* group showed a significantly higher fungal load than the *ups1*∆/∆ group.

We assessed the impact of *UPS1* on *C. albicans* pathogenicity using a mouse vulvovaginal infection model. Before inoculation, we subcutaneously injected estradiol benzoate into the mice to induce their estrus. Subsequently, cells of three strains (WT, *ups1*∆/∆, and *ups1*∆/∆*: UPS1*) were inoculated into the mice’s vaginas for infection. On the 7th day post-infection, the vaginal orifices, vaginas, and vaginal lavage fluids of the mice were examined and analyzed. The results (Figure 5C) showed that: mice in the WT infection group exhibited obvious inflammatory symptoms at the vulvar vaginal orifice, manifested as redness, swelling, and ulcers; mice in the *ups1*∆/∆ infection group had relatively normal-appearing vaginal orifices, with only partial swelling observed; mice in the *ups1*∆/∆*: UPS1* infection group showed significant redness and swelling at the vaginal orifice. Hematoxylin-Eosin (HE) staining showed that the control group’s mice had intact vaginal mucosa with a well-developed superficial stratum corneum. In the wild-type infection group, the stratum corneum was absent, with squamous epithelial cell proliferation and extensive inflammatory cell infiltration. The *UPS1∆/∆* group retained some stratum corneum, and inflammatory cell infiltration was notably reduced. Compared with the *ups1*∆/∆ group, the *ups1*∆/∆*: UPS1* group showed more severe vaginal tissue damage (Figure 5D).

Gram staining of vaginal lavage fluid showed that the *ups1*∆/∆ group had a significantly lower amount of *C. albicans* than the WT group, with no fungal attachment to epithelial cells of the vaginal mucosa. In contrast, both the WT and *ups1*∆/∆*: UPS1* groups developed elongated and thin hyphae that bound to vaginal mucosal epithelial cells resulting in the aggregation of dense pathogenic fungi (Figure 5D). Furthermore, vaginal lavage fluid was cultured on dishes to assess the fungal load in the mouse vaginal cavity (Figure 5E). The results indicated that the *ups1*∆/∆group had a significantly lower fungal burden compared to the WT group, whereas the *ups1*∆∆*: UPS1* exhibited a markedly higher fungal burden than the *ups1*∆/∆ strain. As illustrated in Figure 5F, relative to the blank group, the WT group displayed a marked elevation in serum pro-inflammatory cytokine levels accompanied by a reduction in anti-inflammatory cytokine concentrations. On the contrary, the *ups1*∆/∆ mutant strain presented an opposing trend. The *ups1*∆/∆*: UPS1* complemented strain reversed these changes, restoring the cytokine levels to those detected in the WT group. These results indicate that knockout of the *UPS1* gene substantially weakens the pathogenicity of *C. albicans.* We further detected the transcriptional abundances of the three inflammatory factors in each group via quantitative real-time polymerase chain reaction (qRT-PCR). The outcomes obtained were highly consistent with the data derived from the ELISA (Figure 5G). Collectively, these observations suggest that knockout of the *UPS1* gene compromises the ability of *C. albicans* to establish an infection in the host.

## 4. Discussion

Mitochondria are core organelles regulating virulence factor synthesis in pathogenic fungi, but the specific role of the mitochondrial lipid transporter *UPS1* in *C. albicans* remained unclear prior to this study. To address this gap, we constructed a *UPS1* deletion mutant (*ups1*∆/∆) via homologous recombination and a complementation strain using CRISPR-Cas9-mediated knock-in, with reverse screening to ensure result reliability. Functional experiments, transcriptome sequencing combined with qRT-PCR, and in vitro/in vivo infection models showed that *UPS1* deletion induces significant mitochondrial dysfunction (reduced ATP, MMP, and excessive ROS), impairs carbon source utilization and stress tolerance, downregulates hyphal/adhesion genes (ECE1, ALS1, ALS3), upregulates ERG11, and ultimately reduces strain adhesion, biofilm formation, and virulence. These findings confirm that *UPS1* regulates *C. albicans* growth, virulence, and host–pathogen interactions by modulating mitochondrial function.

To clarify *UPS1*’s unique role in *C. albicans* mitochondrial regulation, we compared it with our group’s previously studied mitochondrial proteins OCT1 and NUM11. Like *UPS1*, the protease OCT1 modulates *C. albicans* virulence by maintaining mitochondrial homeostasis [9,38], but *UPS1* is more azole-sensitive, with its deletion upregulating ERG11 to enhance azole tolerance trait not observed in OCT1. In contrast, the anchor protein NUM11 reduces pathogenicity via regulating mitochondrial homeostasis and cell wall structure, while *UPS1* acts as a lipid transporter that forms a heterodimer with Mdm35 to mediate PA transport for CL synthesis and maintain mitochondrial function [39]. These mechanistic differences indicate that *UPS1*, OCT1, and NUM11 regulate *C. albicans* growth and pathogenicity via distinct pathways (lipid transport, protein quality control, and mitochondrial anchoring, respectively), forming a coordinated regulatory network [10]. Additionally, *C. albicans UPS1* is evolutionarily conserved with Saccharomyces cerevisiae *UPS1* (both Mdm35-dependent for PA/CL transport), but species-specific differences exist S. cerevisiae *UPS1* deletion mainly affects mitochondrial cristae and Mgm1 expression, while *C. albicans UPS1* deletion additionally regulates virulence and drug tolerance, consistent with its role as an opportunistic pathogen [40,41].

The findings of this study demonstrate that *UPS1* exerts a central regulatory role in *C. albicans* by modulating mitochondrial function, which serves as the core hub linking *UPS1* to the fungi’s biological and pathogenic traits. Specifically, *UPS1* is essential for maintaining mitochondrial homeostasis, as its role in mediating PA transport and subsequent CL synthesis is critical for preserving mitochondrial structural integrity and respiratory chain functionality. This regulatory effect of *UPS1* on mitochondria further translates to broader impacts on *C. albicans*’ environmental adaptability, including its ability to utilize carbon sources and tolerate temperature stress, highlighting *UPS1*’s significance in the fungus’s survival in diverse host microenvironments. Additionally, the results indicate that *UPS1* is involved in modulating cell membrane and cell wall homeostasis, as well as drug tolerance, through its influence on ergosterol synthesis pathways. Most importantly, the data confirms that *UPS1* is a key regulator of *C. albicans* virulence, as its modulation of mitochondrial function is indispensable for the development of core virulence traits and the fungus’s ability to establish successful infections in host models. Collectively, these observations underscore that *UPS1* acts as a multifunctional regulator in *C. albicans*, integrating mitochondrial function with the fungus’s metabolic adaptation, stress response, and pathogenic potential.

This study has certain limitations. Mechanistically, the specific molecular pathways linking *UPS1* deletion-induced CL synthesis dysfunction to mitochondrial respiratory chain abnormalities remain unclear [42]; additionally, whether *UPS1* directly or indirectly regulates ERG11 (e.g., via specific transcription factors) has not been fully elucidated. The potential interactions between *UPS1*, *OCT1*, and *NUM11* also require further verification. Experimentally, only two infection models were used in this study, which limits insights into the role of *UPS1* in *C. albicans* systemic infections. In drug tolerance studies, we only focused on azole drugs and several common cell membrane/cell wall stressors, excluding other types of antifungal drugs (e.g., echinocandins), resulting in a relatively limited research scope. Furthermore, transcriptome analysis only validated the expression of a few key genes, potentially missing other important pathways regulated by *UPS1*.

Despite the limitations, this study systematically clarifies the biological function of *UPS1* in *C. albicans*, filling the research gap in the role of *UPS1* in opportunistic pathogens. By comparing *UPS1* with OCT1 and NUM11, we further improved the mitochondrial regulatory network of *C. albicans* and enhanced the understanding of the role of mitochondria in fungal pathogenicity, while revealing the unique role of *UPS1* in regulating drug tolerance, which enriches the research on fungal drug resistance mechanisms. *UPS1* is a potential target for new antifungal drugs, as its deletion significantly reduces *C. albicans* virulence and alters its drug tolerance. Future research could design specific *UPS1* inhibitors to disrupt mitochondrial homeostasis of *C. albicans*, thereby reducing its pathogenicity and drug tolerance, and providing new drug candidates for the clinical treatment of *C. albicans* infections. Meanwhile, the regulatory effect of *UPS1* on ERG11 found in this study provides a theoretical basis for optimizing the treatment plan of existing azole drugs; co-regulating the expression of *UPS1* and ERG11 can improve the efficacy of azole drugs and reduce the generation of drug-resistant strains. In addition, the research methods and ideas of this study can also provide a reference for the functional research of mitochondrial lipid transporters in other pathogenic fungi.

## Data Availability

The original contributions presented in this study are included in the article/Supplementary Material. Further inquiries can be directed to the corresponding authors.

## Supplementary Materials

The following supporting information can be downloaded at: https://www.mdpi.com/article/10.3390/jof12060411/s1, Figure S1. Identification of *UPS1* gene deletion in *C. albicans*. (A) Upstream and downstream regions of the UPS1 gene, along with screening marker fragments for LEU2 and HIS1. (B) Fused upstream and downstream sequences of the UPS1 gene with corresponding screening markers. (C) Identification of the UPS1Δ∷LEU2 strain via Lasso PCR. (D) Detection of the *ups1Δ/Δ* strain using PCR kits. (E)CRISPR-mediated reconstitution of the UPS1 gene into the knockout strain, resulting in three fragments: A (Cas9 and sgRNA for inducing double-strand breaks at the UPS1 locus), B (vector plasmid), and C (target gene UPS1). (F) Identification of the restored mutant strain *UPS1Δ/Δ: UPS1*; Figure S2. Regarding the growth of various strains in liquid culture medium over 2 hours; Figure S3. To investigate whether ALS3 and ECE1 gene expression is regulated, RNA was extracted from each strain under mycelial culture conditions and analyzed by RT-qPCR. The knockout strain ups1∆/∆ exhibited downregulated expression of ALS3 and ECE1 genes, with more pronounced effects.; Table S1. *Candida albicans* strains used in this study; Table S2. The primers used in this study; Table S3. Plasmids used in this study; Table S4. The primers used in qPCR; Table S5. Transcriptome data of UPS1Δ/Δ strain compared with the WT strain.
