# Supplementary material for: The Role of Mitochondrial Protein UPS1 in Regulating Pathogenicity of Candida albicans
Source: J Fungi (Basel). 2026 Jun 4;12(6):411. doi: 10.3390/jof12060411 (PMC13302225; doi:10.3390/jof12060411)
Supplement: Supplementary file 1 [file jof-12-00411-s001.zip › jof-4290700-supplementary.pdf]

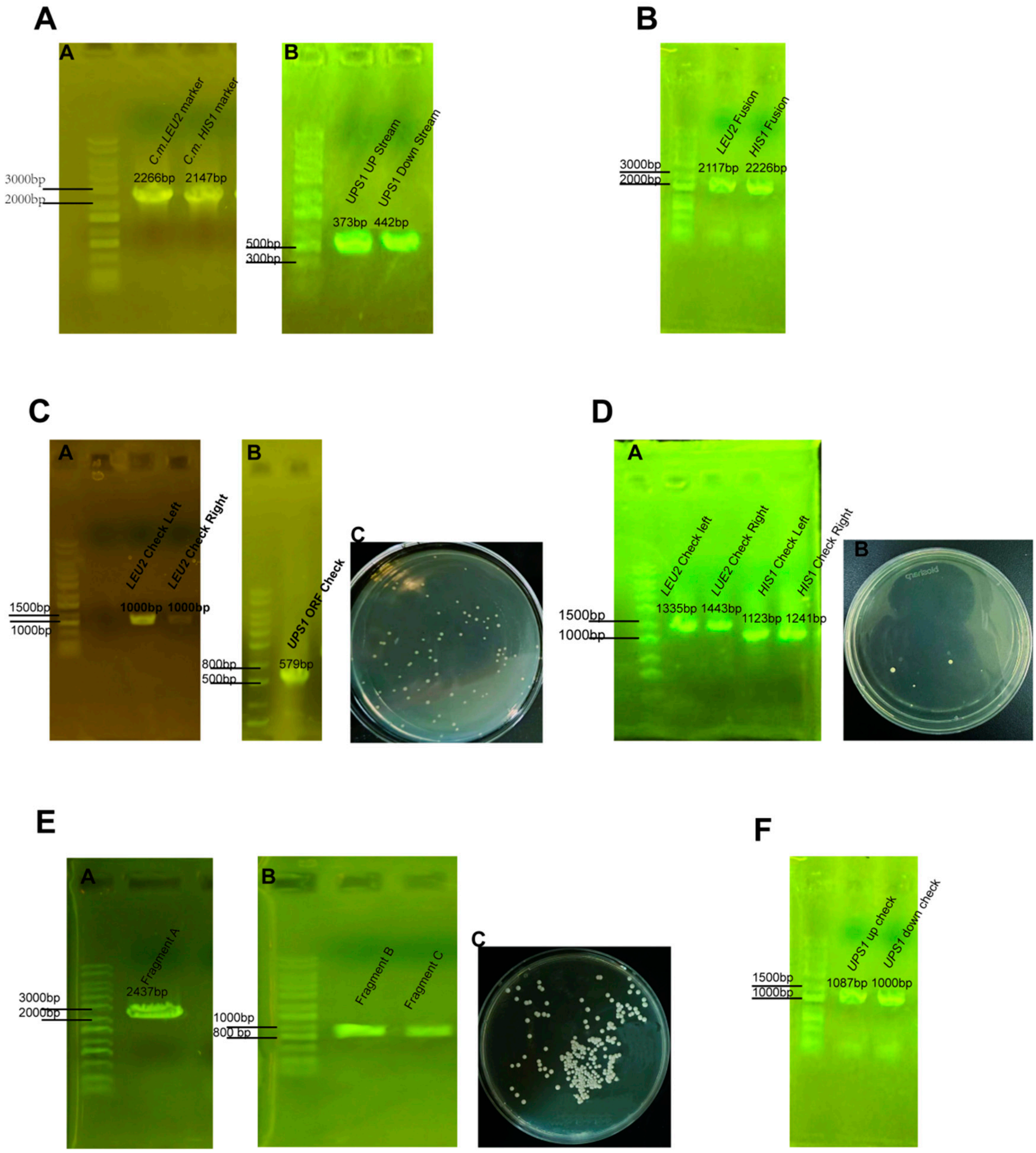

Figure S1: Identification of *UPS1* gene deletion in *C. albicans*. (A) Upstream and downstream regions of the UPS1 gene, along with screening marker fragments for LEU2 and HIS1. (B) Fused upstream and downstream sequences of the UPS1 gene with corresponding screening markers. (C) Identification of the UPS1Δ::LEU2 strain via Lasso PCR. (D) Detection of the *ups1Δ/Δ* strain using PCR kits. (E)CRISPR-mediated reconstitution of the UPS1 gene into the knockout strain, resulting in three fragments: A (Cas9 and sgRNA for inducing double-strand breaks at the UPS1 locus), B (vector plasmid), and C (target gene UPS1). (F) Identification of the restored mutant strain *UPS1Δ/Δ: UPS1*.

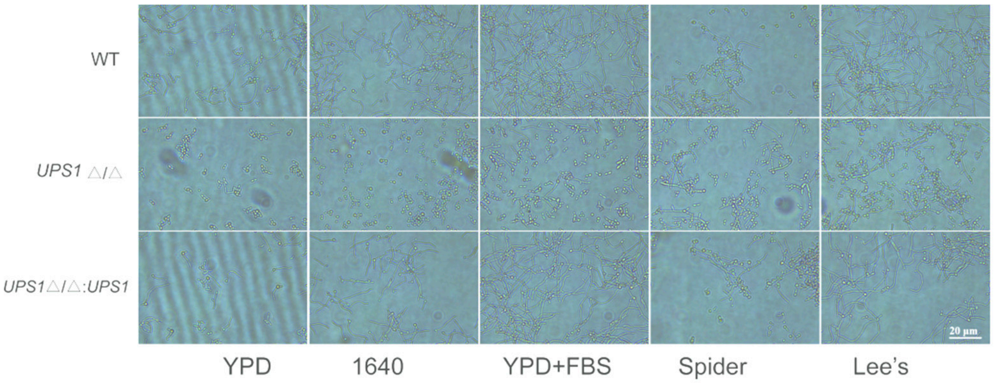

Figure S2：Regarding the growth of various strains in liquid culture medium over 2 hours, it is evident that the knockout strains exhibit extremely slow growth in the medium. Even in FBS and Sprider media capable of inducing hyphal growth, they maintain a yeast-like state with only minimal pseudohyphal formation.

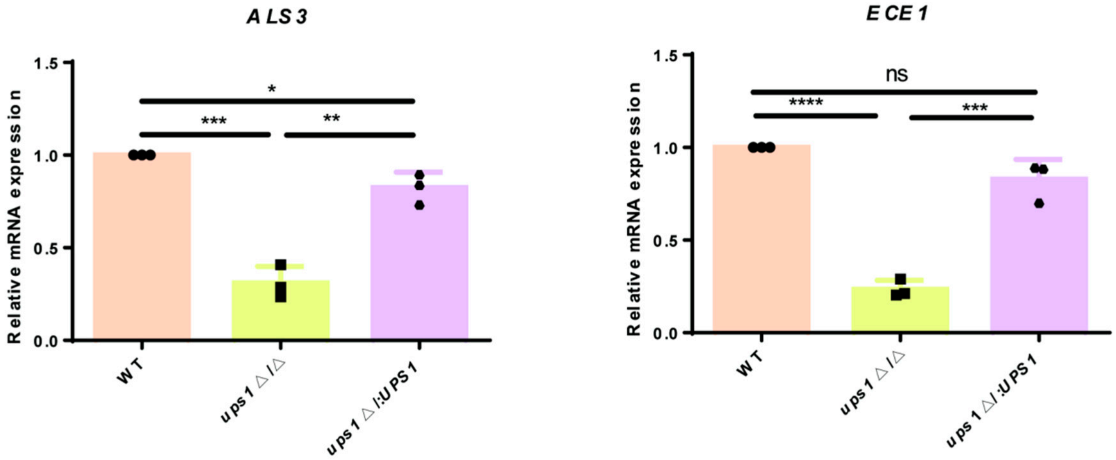

Figure S3: To investigate whether ALS3 and ECE1 gene expression is regulated, RNA was extracted from each strain under mycelial culture conditions and analyzed by RT-qPCR. The knockout strain ups1Δ/Δ exhibited downregulated expression of ALS3 and ECE1 genes, with more pronounced effects.

S1

Professor Jiang Yuanying (Center for New Drug Research, School of Pharmacy, Second Military Medical University) donated the wild-type strain SN152 and plasmids pSN40 and pSN52 to our research group (Zhu et al. 2024).

Table S1. *Candida albicans* strains used in this study

Strain	Genotype*	Parent	Reference
SN152 (wild type strain)	*arg4Δ/arg4Δ leu2Δ/leu2Δ his1Δ/his1Δ URA3/ura3Δ∷ imm^{434} IRO1/iro1Δ∷imm^{434}*	SC5314	Noble[1]
UPS1Δ∷LEU2	*UPS1/UPS1Δ ∷ C.m.LEU2 arg4Δ/arg4Δ leu2Δ/leu2Δ*	SN152 (wild type	This study

	his1Δ/his1Δ URA3/ura3Δ∷imm^{434} *IRO1/iro1Δ∷imm*^{434}	strain)	
UPS1Δ/Δ	*UPS1Δ ∷ C.m.LEU2/num11Δ ∷ C.d.HIS1 leu2Δ/leu2Δ his1Δ/his1Δ URA3/ura3Δ∷imm*^{434} *IRO1/iro1Δ∷imm*^{434}	SN152 (wild type strain)	This study
UPS1Δ/Δ: UPS1	*UPS1Δ ∷ C.m.LEU2/num11Δ ∷ C.d.HIS1 leu2Δ/leu2Δ his1Δ/his1Δ URA3/ura3Δ∷imm*^{434} *IRO1/iro1Δ∷imm*^{434} *RPS1/rps1*	*UPS1Δ/Δ*	This study
UPS11-mNeonGreen	arg4Δ/arg4Δ leu2Δ/leu2Δ his1Δ/his1Δ URA3/ura3Δ∷ imm^{434} IRO1/iro1Δ∷imm^{434} UPS1::mNeonGreen-NAT	SN152	This study

* *C.m.*, *Candida maltosa*; *C.d.*, *Candida dubliniensis*.

1. Noble SM, Johnson AD. Strains and strategies for large-scale gene deletion studies of the diploid human fungal pathogen *Candida albicans*. *Eukaryot Cell.* 2005;4(2):298-309.

Table S2. The primers used in this study.

Primer Name	Sequence (5' to 3')
LEU2 Check Left	AGAATTCCCAACTTTGTCTG
LEU2 Check Right	AAACTTTGAAC CCGGCTGCG
HIS1 Check Left	ATTAGATACGTTGGTGGTTC
HIS1 Check Right	AACACAACTGCACAATCTGG
UPS1 P1	AAATTCTTGAAATAACAGTC
UPS1 P2	TTAATCACATTGACTTGGTG
AHO1096	GACGGCACGGCCACGCGTTTAAACCGCC
AHO1098	CAAATTAAAAATAGTTTACGCAAG
HIS LEUpOUT 1	CGTAAACTATTTTTAATTTGTTGACAAGAAACATATTGAGATTTTAGAGCTAGAAATAGC
UPS1 check up	CCCAGTCCTTTTGATGACTT
UPS1 check down	GCTTTAGCTTTCTC
UPS1 mNeonGreen Long up P1	AGTAATGGATTATTATTACAAGAGAGAAAGCTAAAGCCAAAGCCACCAAGTCAATGTGATatggtttctaaaggtgaaga
UPS1 mNeonGreen Long down P1	GTTACTAATATATTATCATTCTACTTTAATGTTATTCTGTTAATTTCTTTAGTAACGCTTtctagaactagtggatctga

Table S3. Plasmids used in this study

Plasmid	Genotype	Reference
pSN40	*C.m.* with *LEU2* screening marker, Kana^r	Noble[1]
pSN52	*C.d.* with *HIS1* screening marker, Kana^r	

1. Noble SM, Johnson AD. Strains and strategies for large-scale gene deletion studies of the diploid human fungal pathogen *Candida albicans*. *Eukaryot Cell.* 2005;4(2):298-309.
2. Dennison PM, Ramsdale M, Manson CL, Brown AJ. Gene disruption in *Candida albicans* using a synthetic, codon-optimised Cre-loxP system. *Fungal Genet Biol.* 2005;42(9):737-748.

Table S4. The primers used in qPCR.

Gene	Forward primer (5' to 3')	Reverse primer (5' to 3')
ALS1	TTGGGTTGGTCCTTAGATGG	ATGATTTCAAAGCGTCGTTC
ALS3	CCTATACCACTGCTACTACCGTTA	GTATGGTTGGTGTAATGAGGACG
ERG11	GGGATACTGCTGCTGCCAAAG	GAACGCACCGATGAAAGCACAAC
FTRI	GGTGGTAGTTCCGACCCTCAATTG	GCCATCATCCACCATGCTCCAG
ECE1	GTATGGTTGGTGTAATGAGGACG	CAGGAACAGTAGGTGCTTGGTCAG
IL-6	GAAACCGCTATGAAGTTCCTCTCTG	GTATCCTCTGTGAAGTCTCCTCTCC
TNF-α	AAGACACCATGAGCACAGAAAGC	GCCACAAGCAGGAATGAGAAGAG
IL-6	TTCGGTCCAGTTGCCTTCTCC	TCTGAAGAGGTGAGTGGCTGTC

All primers were designed by SnapGene Viewer software.

Table S5. Transcriptome data of *UPS1*Δ/Δ strain compared with the WT strain.

Accession to cite for these SRA data:PRJNA1102310
